# Attitudes and compliance with the WHO surgical safety checklist: a survey among surgeons and operating room staff in 138 hospitals in China

**DOI:** 10.1186/s13037-020-00276-0

**Published:** 2021-01-06

**Authors:** Jie Tan, James Reeves Mbori Ngwayi, Zhaohan Ding, Yufa Zhou, Ming Li, Yujie Chen, Bingtao Hu, Jinping Liu, Daniel Edward Porter

**Affiliations:** 1 School of Clinical Medicine, Tsinghua University, Beijing, 100084 China; 2 Medical Department, Linyi Tumour Hospital, Linyi, 276002 China; 3 Anaesthesiology Department, Ningbo Medical Center Lihuili Hospital, Ningbo, 315040 China; 4 Department of Trauma and Bone Tumour, Tongji Hospital, Tongji University, Shanghai, 200065 China; 5 Department of Spine Surgery, Luohe Central Hospital, Luohe Medical College, Luohe, 462000 China; 6 Operating Department, The First Hospital of Tsinghua University (Beijing Huaxin Hospital), Beijing, 100016 China; 7 Department of Orthopaedic Surgery, The First Hospital of Tsinghua University (Beijing Huaxin Hospital), Beijing, 100016 China

**Keywords:** WHO safety checklist, Compliance, China

## Abstract

**Background:**

Ten years after the introduction of the Chinese Ministry of Health (MoH) version of Surgical Safety Checklist (SSC) we wished to assess the ongoing influence of the World Health Organisation (WHO) SSC by observing all three checklist components during elective surgical procedures in China, as well as survey operating room staff and surgeons more widely about the WHO SSC.

**Methods:**

A questionnaire was designed to gain authentic views on the WHO SSC. We also conducted a prospective cross-sectional study at five level 3 hospitals. Local data collectors were trained to document specific item performance. Adverse events which delayed the operation were recorded as well as the individuals leading or participating in the three SSC components.

**Results:**

A total of 846 operating room staff and surgeons from 138 hospitals representing every mainland province responded to the survey. There was widespread acceptance of the checklist and its value in improving patient safety.

860 operations were observed for SSC compliance. Overall compliance was 79.8%. Compliance in surgeon-dependent items of the ‘*time-out*’ component reduced when it was nurse-led (*p* < 0.0001). WHO SSC interventions which are omitted from the MoH SSC continued to be discussed over half the time. Overall adverse events rate was 2.7%. One site had near 100% compliance in association with a circulating inspection team which had power of sanction.

**Conclusion:**

The WHO SSC remains a powerful tool for surgical patient safety in China. Cultural changes in nursing assertiveness and surgeon-led teamwork and checklist ownership are the key elements for improving compliance. Standardised audits are required to monitor and ensure checklist compliance.

## Introduction

The World Health Organization (WHO) launched the Safe Surgery Saves Life campaign in January 2007 with the aim of improving consistency in surgical care and adherence to safety practices. In June 2008 the WHO Surgical Safety Checklist (SSC) was published to help operating room staff improve teamwork and ensure the consistent use of safety processes [1]. Use of the WHO surgical safety checklist (SSC) is associated with a significant decrease in postoperative complication (30%) and mortality rates [2], improved compliance with standard processes of care and better quality of teamwork in the operating room [1]. Other benefits which have been reported following implementation of the checklist include cost savings [3]. The WHO SSC has become one of the most significant and widely used innovations in surgical safety of the past 20 years.

In the WHO Guidelines for Safe Surgery 2009, it is written ‘*This checklist is not intended to be comprehensive. Additions and modifications to fit local practice are encouraged*’ [4]. As a member of the World Alliance for Patient Safety, the Chinese Ministry of Health (MoH) has devoted long-term administrative efforts to implement the SSC albeit after significant modification, for example by increasing number of items from 22 to 33. Although most element of the WHO SSC remain part of the officially designated MoH SSC there are sufficient differences to warrant comment. The three components of the SSC, colloquially known as ‘*Sign–In’*, ‘*Time-Out*’ and ‘*Sign-Out*’ all remain as key checklist components in the MoH SSC. However, five items found in the WHO SSC are removed completely. Although some deleted items are included as part of other formal checklists (for example *‘difficult airway and aspiration risk*’ is part of a separate pre-operative anaesthetic check-list), these do not mandate that problems should be discussed with other team-members. Some single items in the WHO SSC which include multiple checks have been split (for example ‘*has the patient confirmed his/her identity, site, the procedure and consent?*’ becomes four separate items in the MoH SSC). Finally, there are several additional interventions found mainly in ‘*sign-in*’ and ‘*sign-out*’ components. These differences are highlighted in Table 1. Since March 2010 the MoH stipulated that implementation of their SSC would be one of the core measurements for assessing hospital performance. However, uptake of the MoH SSC among surgical teams remained low in a survey led by Peking Union Medical College Hospital (PUMCH) in 2012 which revealed that full completion rates were only 84.7, 55.1 and 33.1% at each of the three successive checklist stages [5]. PUMCH attempted a further revision in 2015 with items reduced to 22 [6]. Following a re-implementation programme carried out at four hospitals between June and December 2015 their completion rates of all components improved to over 80% [6]. However as of 2020 the 33 item MoH SSC remains the officially designated checklist. Yet no Chinese publication or report can be referenced to describe the process of creating the MoH SSC. In contrast, the *WHO Guidelines for Safe Surgery 2009* explicitly follow WHO recommended steps in technical guideline development, including detailed documentation of the process of guideline development [4].

**Table 1:** Different versions of the Surgical Safety Checklist (SSC) (items adjacent to each other in the two checklists have equivalence)

Sign-in		Time-out		Sign-out	
WHO SSC	MoH SSC	WHO SSC	MoH SSC	WHO SSC	MoH SSC
Has the patient confirmed his/her identity, site, the procedure and consent	Confirm the patient’s name, gender, age	Confirm all team members have introduced themselves by name and role			Confirm the patient’s name, gender, age
	Confirm the procedure	Confirm the patient’s name, procedure and where the incision will be made	Confirm the patient’s name, gender, age	The name of the procedure	Confirm the name of the procedure
	Surgery consent		Confirm the procedure		Confirm the usage of the drug and blood transfusion
	Anesthesia consent		Confirm the site and is the site marked?	Completion of instrument, sponge and needle counts	Completion of instrument, sponge and needle counts
Is the site marked	Confirm the site and is the site marked?	What are the critical or non-routine steps	What are the critical or non-routine steps	Specimen labelling	Specimen labelling
Is the anesthesia machine and medication check complete	Confirm the type of anesthesia	How long will the case take	How long will the case take		Skin condition
Is the pulse oximeter on the patient and functioning	Check the anesthesia machine, including putting on the pluse oximeter	What is the anticipated blood loss	What is the anticipated blood loss		Any IV tubes, gastric tube, urinary catheter, or any other tubes
Known allergy	Known allergy	Are there any patient-specific concerns	Are there any patient-specific concerns		Where will the patient be transferred to
Difficult airway or aspiration risk		Has sterility been confirmed	Has sterility been confirmed	Whether there are any equipment problems to be addressed	
Risk of >500 ml blood loss		Are there equipment issues or any concerns	Are there equipment issues or any concerns	What are the key concerns for recovery and management of the patient	
	Skin condition	Has antibiotic prophylaxis been give within the last 60 min	Drug administration pre and intro-operation		
	Skin prepartion before surgery	Is essential imaging displayed	Is essential imaging displayed		
	IV line establishment				
	Skin test result of antibiotics				
	Preparation of blood products				
	Prothesis/implant/imaging				

*WHO* World Health Organsiation. *MoH* Chinese Ministry of Health

Emerging evidence suggests that the use of the safety checklist in practice is unreliable and that Operating Room teams display significant variation in how they use the tool [7]. Observational studies of surgical ‘*time-outs’* and ‘*sign-outs’* in a number of countries, including the US, UK, Switzerland, and Australia have concluded that required checks are often only partially completed or completed in an abbreviated manner, team members are frequently absent during the checks, or they often fail to actively participate [8–11]. There is little or no objective evidence as to how widespread such problems of compliance might be, although the effectiveness of the checklist as a safety tool is thought to be affected by poor planning and haphazard introduction methods [10].

Ten years after introduction and implementation of the MoH SSC, the purpose of this study was to explore how elements of the WHO Surgical Safety Checklist are being used in practice in China by observing all three checklist components during elective surgical procedures. In particular we wished to investigate which items in the WHO SSC continue to be discussed as part of the safety culture in operating suites in China even if they are absent from the MoH SSC. In this study, we also sought to understand the attitudes and perceptions of the professionals using the Surgical Safety Checklist.

## Methods

We developed a questionnaire for operating room staff and surgeons based on other research in this area [12, 13] and which would be delivered through a ubiquitous social media platform. Number of questions was predetermined to be less than 20 (estimated completion time 7–8 min) since on-line survey abandon rates increase significantly beyond this point [14]. Specifically, our survey consisted of four domains: 1. Questions 1–4 represented basic and demographic information about the responder’s role and hospital level. 2. For teamwork and safety environment, the Safety Attitudes Questionnaire (SAQ) is a validated instrument used to measure attitudes and perceptions in various safety-related domains in healthcare [15]. A modification has been developed for use in the operating rooms in which six items relating to teamwork and safety climate are relevant to checklist intervention [13]. 3. For attitudes towards the checklist**,** the same published survey instrument had designed six additional items specifically related to the responder’s opinion of the checklist [13] and these were also included in our survey. 4. The survey included two questions about staff perception and observation of patient anxiety due to repetitive checks of their identity and potential problems during surgery and were previously used in a European survey of patients’ attitudes [12]. All responses were recorded on a five-point Likert scale (1 = disagree strongly, 2 = disagree slightly, 3 = neutral, 4 = agree slightly, 5 = agree strongly). At the end of the questionnaire, we included an open comments question to explore unstructured opinions.

The survey was sent to operating room staff and surgeons over a 4-week period from 1 May 2020 to 29 May 2020. Responses were accepted up to end of July 2020. An online network of Chinese national surgical conference attendees (2019) as well as surgical, anaesthetic and nursing trainee networks (2016–2020) were engaged via the social media platform WeChat®. Onward dissemination of the survey among hospital operating room colleagues was encouraged.

We conducted a prospective observational study at five Level 3 hospitals in four provinces (Beijing, Shandong, Zhejiang and Henan). These hospitals were part of an informal training network for medical personnel. Hospital compliance-rates with the surgical safety checklist was unknown prior to the study. As in previously published methodology, each hospital identified one operating room to serve as the study room [1]. The focus of this study was entirely on observations of operating room staff behaviour and not on the patient. Hence institutional ethical approval was not a requirement for this study.

A local data co-ordinator was chosen at each site who was also the observer (2 anaesthetists, 2 nurses and 1 surgeon). These underwent training to enable them to document specific item performance via a combination of observation and occasional verbal confirmation with the surgical team. The observer had no other clinical responsibilities at the study site during the period of observation and was not empowered to intervene if an item was not performed. Only if all three sections of the WHO surgical safety checklist were observed could the case be included in this study. Data collected included surgical specialty and staff compliance with specific WHO SSC items.

The presence, absence or lack of engagement of key clinical staff during each stage was also documented during each of the three components, with additional observations of operating room staff behaviour during the ‘*timeout’* component. An earlier study of SSC checklist compliance in China recorded overall staff attitudes as ‘attentive’, ‘hasty’, ‘casual’ or ‘missing’ [6]. We wished to record actual engagement of individual professionals with the aim of discovering if these could illuminate the cause of poor compliance with specific SSC items. During the critical ‘*time-out*’ component the role of the professional who led the process was recorded, as well as whether each professional took an active part in the process and whether they stopped what they were doing during the process.

Adverse events were defined as events that resulted in an operative delay due to check-list related identification of problems with instruments, pre-operative medication or central venous access [16]. All adverse events were documented during the operative procedure.

We targeted a cohort of 1000 completed SSC observations. Each site accumulated 200 observations from January to June 2020 except for site B where the observer was unavoidably assigned to other duties during this period. Therefore total observations were for 860 SSCs.

### Statistical analysis

Data were analysed using IBM SPSS® Statistics 26.0. Descriptive statistics (frequencies and percentages) were calculated to demonstrate the overall quality of checklist use, analysed by hospital (Sites A to E). Chi-square (+/− Yates correction) was used to assess significant associations between hospital for any of the categorical variables assessed. For all analyses, significance was set at *p* < 0.05.

## Results

A total of 846 individuals from 138 hospitals representing all mainland Chinese provinces responded to the survey (Table 2). 74.3% were working in a Level 3 hospital (most advanced level), 15.5% in a Level 2 hospital, 1.9% in a Level 1 hospital and 8.3% in private hospitals. We obtained responses from staff with a representative distribution of roles in the SSC process (332 surgeons (39.2%), 299 anaesthetists (35.3%) and 215 operating room nurses (25.4%)).

**Table 2:** Survey questionnaire results from Chinese operating room staff and surgeons

Question number	Basic and Demographic information						
1	What is the name of your hospital	*N* = 138 hospitals					
2	What level of hospital do you work at	Level 3: 74.3%	Level 2: 15.5%	Level 1: 1.9%	Private: 8.3%		
3	Please state your profession	Theatre Nurse: 25.4%	Anesthetist: 35.3%	Surgeon: 39.2%			
4	Gender	Male: 54.1%	Female: 45.9%				
	Teamwork and Safety environment	Strongly disagree	Disagree	Neither agree or disagree	Agree	Strongly agree	Teamwork and Safety environment score
5	I would feel safe being treated here as a patient	4.0%	2.2%	5.7%	39.0%	49.1%	4.27
6	Briefing OR personnel before a surgical procedure is important for patient safety	3.3%	4.8%	6.7%	34.0%	51.1%	4.25
7	I am encouraged by my colleagues to report any safety concerns I may have	1.8%	4.2%	10.3%	48.5%	35.3%	3.84
8	In the opearitng room here, it is difficult to speak up if I perceive a problem with patient care	33.1%	42.4%	12.8%	7.2%	4.5%	3.92
9	The physicians and nueses here work together as a well-co-ordinated team	0.9%	5.1%	6.0%	50.0%	37.9%	4.19
10	Personnel frequently disregard rules or guidelines that are established for the OR	36.2%	35.0%	13.6%	11.5%	4.0%	3.89
	Attitudes towards the checklist	Strongly disagree	Disagree	Neither agree or disagree	Agree	Strongly agree	
11	The checklist was easy to use	2.7%	2.8%	15.7%	51.8%	27.0%	
12	The checklist took a long time to complete	19.4%	49.4%	18.6%	9.1%	3.6%	
13	The checklist improved operating room safety	0.8%	0.8%	7.9%	42.9%	47.5%	
14	Communication was improved through use of the checklist	0.8%	2.0%	11.6%	52.0%	33.6%	
15	The checklist helped prevent errors in the operating room	2.5%	2.0%	6.0%	46.7%	42.8%	
16	If I were having an operation, I would want the checklist to be used	1.9%	1.5%	5.7%	42.1%	48.8%	
	Staff perception and observation of patient anxiety	Strongly disagree	Disagree	Neither agree or disagree	Agree	Strongly agree	
17	Do you think that a conscious patient may become anxious if we repetitively confirm the patient’s identity, the procedure and operation site and discuss the potential airway problems and blood loss in their hearing	10.0%	20.2%	28.8%	33.8%	7.1%	
18	Have you ever experienced a patient becoming anxious because of the issue above	Yes: 51.8%	No: 48.2%				

The mean safety attitude score was 4.06 (minimum score = 1, maximum score = 5). Negative questions were reverse-scored. The four items asking a positive question scored well, with mean 85.6% affirming their environment as safe and collegiate, however the two negative items ‘*it is difficult for me to speak up*’ and ‘*personnel frequently disregard the rules*’ scored less well with mean 73.4% disagreeing with these statements (X^2^ = 42.50, *p* < 0.0001). 88.1% of all responders stated that they would feel safe being treated there as a patient.

Following its universal implementation from 2010 it was assumed that the MoH SSC was the official version used in all hospitals. For the domain ‘attitudes towards the checklist’, only 12.7% of responders deemed that the checklist ‘*took a long time to complete*’. 78.8% agreed it was ‘*easy to use*’. A large majority agreed that the checklist improved operating room safety and communication (90.4 and 85.6% respectively) and 89.5% thought that the checklist helped prevent errors in the operating room. Only 3.4% disagreed with the statement that they would want the checklist used if they were having an operation.

Questions about anxiety induced in patients revealed that over 40% considered that a conscious patient might become anxious during repetitive confirmation of her/his identity, the procedure and operation site, or discussion of potential airway hazards and blood loss in their hearing. Over half claimed that they had experience of a patient becoming anxious because of this. Furthermore, the most common open comment was that potential blood loss and airway risk should not be discussed in the presence of a conscious patient.

At all sites completion of checklists was paper-based and these were archived in the patient’s medical record after completion of the operation. Complete information was obtained from 860 checklist processes in the five hospitals. Hospital characteristics and cases by surgical specialty are reported in Table 3. Just over half of the cases were orthopaedic, with general surgery, gynaecology and thoracic surgery contributing 16, 12 and 11% respectively.

**Table 3:** Site characteristics, number and specialty of operations for observed checklists. Abbreviations: ENT-Ear, Nose and Throat

Item	Sub-item	Site A	Site B	Site C	Site D	Site E	Total
**No of beds**		760	1600	1200	1850	1390	**6800**
**No. of Operating Rooms**		9	20	45	12	28	**114**
**Hospital level**	General Level	Public, level 3	Public, level 3	Public, level 3	Public, level 3	Public, level 3	**\**
	Star Level	No	No	No	5 star	No	**\**
**No. of surgical checklists observed by specialty**	Elective Orthopaedics	159	18	51	196	10	**434**
	Elective General surgery	21	7	25	\	85	**138**
	Elective Gynecology	9	6	16	\	62	**93**
	Elective Urology	11	\	15	\	3	**29**
	Elective Thoracic Surgery	\	29	31	4	40	**104**
	Elective Cardiac Surgery	\	\	8	\	\	**8**
	Elective Neurology Surgery	\	\	6	\	\	**6**
	Elective Vascular Surgery	\	\	3	\	\	**3**
	Elective ENT Surgery	\	\	45	\	\	**45**
	**Total**	**200**	**60**	**200**	**200**	**200**	**860**

As shown in Table 4, compliance with the sign-in items varies from hospital to hospital, but the five items of the WHO SSC which remain part of the MoH SSC achieved over 95% compliance. The remaining two items which are omitted from the MoH SSC were discussed less than 90% of the time. These were ‘*Difficult airway or aspiration risk*’ (84%) and ‘*Risk of>500ml blood loss*’ (90%).

**Table 4:** Compliance with the WHO Surgical Safety Checklist items

	Item	Site A	Site B	Site C	Site D	Site E	Total
*Sign-in’* phase	Has the patient confirmed his/her identity, site, the procedure and consent	100.0%	100.0%	98.0%	100.0%	100.0%	**99.5%**
	Is the site marked	96.5%	80.0%	97.5%	100.0%	100.0%	**97.2%**
	Is the anesthesia machine and medication check complete	100.0%	100.0%	81.5%	100.0%	100.0%	**95.7%**
	Is the pulse oximeter on the patient and functioning	100.0%	95.0%	96.0%	100.0%	100.0%	**98.7%**
	Known allergy	100.0%	100.0%	95.5%	100.0%	100.0%	**99.0%**
	Difficult airway or aspiration risk	87.5%	0.0%	74.5%	100.0%	99.0%	**84.0%**
	Risk of >500 ml blood loss	97.5%	63.3%	70.5%	100.0%	99.0%	**89.9%**
	**Overall ‘*sign-in’***						**94.8%**
*Time-out’* phase	Confirm all team members have introduced themselves by name and role	2.0%	0.0%	0.0%	6.0%	0.0%	**1.9%**
	Confirm the patient’s name, procedure and where the incision will be made	100.0%	100.0%	79.5%	100.0%	93.0%	**93.6%**
	Has antibiotic prophylaxis been give within the last 60 min	100.0%	100.0%	92.0%	100.0%	100.0%	**98.1%**
	What are the critical or non-routine steps	22.5%	25.0%	62.0%	100.0%	10.0%	**47.0%**
	How long will the case take	25.0%	85.0%	79.0%	100.0%	11.0%	**55.9%**
	What is the anticipated blood loss	23.5%	65.0%	80.5%	100.0%	8.0%	**53.8%**
	Are there any patient-specific concerns	20.0%	60.0%	73.0%	100.0%	100.0%	**72.3%**
	Has sterility been confirmed	100.0%	50.0%	77.5%	100.0%	100.0%	**91.3%**
	Are there equipment issues or any concerns	98.5%	65.0%	77.5%	100.0%	100.0%	**92.0%**
	Is essential imaging displayed	98.0%	85.0%	95.0%	100.0%	100.0%	**97.3%**
	**Overall ‘*time-out’***						**70.3%**
*Sign-out*’ phase	The name of the procedure	97.50%	100%	98%	100%	100%	**98.9%**
	Completion of instrument, sponge and needle counts	100%	100%	96.50%	100%	100%	**99.2%**
	Specimen labelling	99%	75%	97.50%	100%	100%	**97.4%**
	Whether there are any equipment problems to be addressed	83.50%	0%	0%	100%	100%	**65.9%**
	What are the key concerns for recovery and management of this patient	7%	10%	0%	100%	11%	**28.1%**
	**Overall ‘*sign-out*’**						**77.9%**
	Average compliance (all items)	75.3%	66.3%	73.7%	95.7%	78.7%	**79.8%**
	Average compliance (for items in both WHO SSC and MoH SSC)	81.2%	81.4%	86.9%	100.0%	83.6%	**87.4%**
	Average compliance (for items only in WHO SSC)	55.5%	14.7%	29.0%	81.2%	61.8%	**53.9%**
	Proportion of items reaching 100% compliance	8/22	7/22	0/22	21/22	15/22	

*WHO* World Health Organisation, *MoH* Chinese Ministry of Health, *SSC* Surgical Safety Checklist

Different professionals were responsible for leading the ‘sign-in’ component at different sites (Table 5). Depending on local policy checklist leaders were a combination of professionals although at three sites anaesthetists were not involved.

**Table 5:** Percentage compliance with WHO Surgical Safety Checklist roles by profession

		Site A			Site B			Site C			Site D			Site E		
		Doctor	Nurse	Anesthetist	Doctor	Nurse	Anesthetist	Doctor	Nurse	Anesthetist	Doctor	Nurse	Anesthetist	Doctor	Nurse	Anesthetist
Sign in	Who participated in this part?	\	100%	\	100%	100%	\	98%	87%	92%	100%	100%	\	100%	100%	100%
Time-out	Who participated in this part?	97%	98%	93%	100%	100%	100%	90%	86%	93%	100%	100%	100%	100%	100%	100%
	Who led this part?	\	100%	\	\	\	100%	1%	1%	92%	21%	11%	68%	\	100%	\
	Who stopped to do the ‘*time-out’*?	15%	97%	26%	80%	100%	85%	2%	3%	74%	100%	100%	100%	100%	100%	100%
Sign out	Who participated in this part?	86%	100%	7%	100%	100%	\	75%	98%	86%	100%	100%	100%	100%	100%	100%
Was the WHO checklist completed before the patient left the operating room?		95%			80%			99%			100%			100%		

Table 4 shows that the WHO SSC checklist item *‘introducing team members by name and role’* which is not part of the MoH SSC was rarely completed at any site (< 2%). The other nine items all remain part of the MoH SSC. These steps were completed well in some centres but not in others. Five items scored over 90% compliance (patient identification /incision site, confirmation of antibiotic prophylaxis, confirmation of sterility, equipment issues, display of essential imaging). Three items achieved less than 60% compliance (identification of critical / non-routine steps, length of surgery and anticipated blood loss). From Table 4 all professional groups engaged in the time-out process at each site, although overall compliance according to professional groups ranged from 86 to 100%. Leadership role varied (nurses at two sites, and anaesthetists at three sites). Staff engagement in an actual ‘time-out’ (where staff stop what they are doing to listen and participate) varied greatly; two sites achieved total compliance, whereas the other three had very poor engagement from at least one of doctors, nurses or anaesthetists. The ‘*time-out*’ component was not seen to be done at all in 6% of cases at Site C (Table 4).

The WHO Guidelines for Safe Surgery 2009 states that certain information should be sought specifically from the surgeon (critical stages, length of surgery, anticipated blood loss) and from the nurse (sterility and equipment issues) [4]. Surgeon compliance with their responsible items averaged 52.3%, whereas nurse compliance averaged 91.7%. Surgeons were significantly worse than all other group and nurses significantly better (X^2^ = 735.5 and 744.4 respectively, both *p* < 0.0001).

Table 5 shows levels of engagement from doctors and nurses at different sites during the ‘*time-out*’ component. There was no clear association between doctors or nurses who failed to engage or to stop their activity during the timeout process and lack of compliance with items which are usually within their sphere of knowledge. On the other hand, the three hospitals in which anaesthetists took the lead in completing the checklist showed a significant association with the three surgeon-related items being better performed, in contrast to nurse-led processes in which these items were not done well (X^2^ = 315.6, 433.6 & 426.6 respectively, all *p* < 0.0001).

Table 4 shows that the three items which remained part of the MoH SSC achieved a high rate of compliance (over 97%). However, the two items encompassing equipment problems and patient recovery plans and concerns were discussed less frequently (66 and 28% respectively).

Participation rates across disciplines were high (Table 5), however anaesthetists were infrequently represented at two of the five sites. Overall, checklists were completed before the patient left the operation suite 97% of the time (range 80–100%).

Overall compliance rates of all 17 items which remained part of the MoH SSC was87% (Table 4). The other five items removed from the MoH SSC were discussed in 54% of cases overall. One hospital (site D) achieved 100% compliance in 21/22 of their checklist items (the next best hospital achieved 100% in 15/22). At site D overall compliance was 96%, significantly better than any of the other hospitals (site D compared with next best site X^2^ = 567.3, *p* < 0.0001).

The incidence of adverse peri-operative events varied among hospitals (Table 6). At most sites overall frequency was less than 3%. However, nearly 30% of cases from site D had missing instruments which led to an operative delay.

**Table 6:** Adverse event categories and incidence (number with percentage in parentheses)

	Site A	Site B	Site C	Site D	Site E	Overall
Number of surgical checklists observed	200	60	200	200	200	**860**
Missing instrumentation leading to intraoperative delay	5(2.5%)	1(1.7%)	2(1%)	48(29%)	11(5.5%)	**67 (7.8%)**
Missing medication before incision	\	\	16(8%)	\	14(7%)	**30(3.4%)**
Broken instruments	2(1%)	\	\	4(2%)	1(0.5%)	**7(0.8%)**
Contaminated instruments	4(2%)	1(1.7%)	3(1.5%)	\	2(1%)	**10 (1.2%)**
Others (difficulty in achieving central venous access)	\	\	2(1%)	\	\	**2 (0.2%)**
**Total**	**11(1.1%)**	**2 (0.7%)**	**23 (2.3%)**	**52 (5.2%)**	**28 (2.8%)**	**116 (2.7%)**

## Discussion

Based on the attitudes survey, operating room staff and surgeons who had used the WHO SSC before hold a generally positive view of the checklist. Only 5.5% of them thought it difficult to use and more than 85% of the OR staff perceived it had value in ensuring patient safety and improving communication. More than 90% of responders claimed that they would want the checklist used for their own care. Even when clinicians express some scepticism about the WHO SSC, the fundamental perception of its value in providing safe care suggests that a well-designed implementation program can be successful in achieving clinician acceptance and use of the checklist [13]. Dixon-Woods, in a recent review of ethnographic studies of the operating room process concluded that major barriers to patient safety were present at both structural and cultural levels [17]. Observational studies have shown an association between good teamwork and decreased risk of postoperative complication [18]. Implementation of a WHO Safe Surgery Saves Lives checklist-based quality improvement project was associated with a small but significant increase in mean teamwork and safety climate score among operating personnel [13]. Positive changes in perception of teamwork and safety climate by these clinicians correlated with the degree of improvement in postoperative morbidity and mortality [13].

Over half of responders said they had seen a patient becoming anxious during use of the checklist, which has not been reported before. It is possible that one reason for poor compliance of staff with these parts of the ‘sign-in’ component is that they would not wish to upset their conscious patient. There is support from a European study that discussion of airway and haemorrhage issues can cause more anxiety than repetitive questions about identity and operative site [12]. It is unfortunate that we cannot identify any published description or rationale for removing items from the MoH SSC, however potentially all five omitted items could induce anxiety if overheard by the patient by highlighting patient risk, an absence of existing strong teamwork, equipment problems or ongoing patient concerns. In contrast, the majority of items added to the MoH SSC do not have these characteristics (Table 1). In China the doctor-patient relationship is often considered tense and fragile [19]. For example, surveys in 2013 found that approximately 70% of patients did not trust physicians [20] and in 2018 that over 60% of obstetricians had experienced a personal lawsuit, and a similar proportion agreed with the concept of practicing defensive medicine [21]. Multiple and well documented reports confirm a high and growing incidence of violence against medical personnel [22], riots, attacks, and protests in hospitals [23] and even cold-blooded murder [24]. It is not surprising that in such an environment every encounter which could induce patient anxiety is scrutinised with the objective of mitigating its negative effects. Whether the checklist interventions which are from the MoH SSC might precipitate real patient concerns and anxieties among Chinese patients remains the subject of further study.

The WHO SSC has been shown to have a beneficial impact on postoperative mortality and morbidity and on team effectiveness in the operating room in a number of studies. Institutions whose frontline workers and managers score higher on safety climate surveys have been found to have lower rates of adverse patient safety indicators as defined by the Agency for Healthcare Research and Quality [25]. Checklists are behavioural interventions; meaning they require a change in the behaviour of the Operating Room team to be effective. The interventions recommended in the WHO SSC are explicitly evidenced [4]. Modification of the checklist to suit local conditions is allowed in the WHO Guidelines for Safe Surgery 2009; since it should not *‘enforce behaviours that the practitioners do not agree with or cannot follow’*. There is a presumption that the additional items found in the MoH SSC are more suitable for the Chinese operating suite environment however despite the lack of explicit evidence. It is also suggested that each component of the SSC should ideally have between 5 and 9 items [4], however the MoH SSC ‘*sign-in*’ component consists of 14 items and the ‘*time-out*’ consists of 11 items. Although one research group published methodological justification for a modified SSC in China [5] this version is not used nationally.

We found that 5 out of 7 items during the ‘*sign-in*’ component achieved over 95% compliance which compares favourably with that of other large studies in China [5, 6]. The remaining two items which are not part of the MoH SSC were questions which the anaesthetist and surgeon would have more direct knowledge than the nurse (difficult airway and anticipated blood loss). Table 4 shows that despite the presence of these clinicians at only 60.4% of ‘*sign-in*’ components, overall discussion rates for these items of the WHO SSC remained over 80%. The WHO Guidelines for Safe Surgery 2009 highly recommends that that ‘*before inducing anaesthesia, the anaesthetist should consider the possibility of large-volume blood loss*’ and explains that ‘*the expected blood loss will be reviewed again by the surgeon before skin incision. This will provide a second safety check for the anaesthetist and nursing staff’.* We found that although the ‘*risk of blood-loss*’ intervention is removed from the sign-in component of the MoH SSC it was still discussed nearly 90% of the time. Perhaps this high figure provides a reason for the relatively low compliance with the same item in the *‘time-out’* component which was only discussed 54% of the time. Whether the physical environment for the pre-anaesthetic ‘sign-in’ component is relevant to difficulties in discussing safety issues is unknown, however the design feature of a separate anaesthetic room adjacent to the operating room is not common in Chinese hospitals.

In the ‘*time-out*’ component, both the MoH SSC and the PUMCH SSC omit the item ‘*introducing team members by name and role*’, the latter giving reason that ‘*it was not necessary for most Chinese procedures as most operating teams are relatively fixed*’. Among all the WHO SSC items this was least performed (less than 2% of ‘time-outs’). When the item is removed from the analysis, other items of the ‘*timeout*’ achieve an average compliance rate of 78%, similar to the 80% compliance-rate achieved by PUMCH in 2015 [6].

The use of anesthetists to lead the ‘*time-out*’ was part of the final SSC protocol in the PUMHC study in 2015 [6], with the authors suggesting that anesthetists ‘*exhibit stronger leadership*’ than the circulating nurse. The possibility that team members other than the circulating nurse may lead the time-out are allowed in the WHO Guidelines for Safe Surgery 2009 report; ‘*The Checklist coordinator can and should prevent the team from progressing to the next phase of the operation until each step is satisfactorily addressed, but in doing so may alienate or irritate other team members. Therefore, hospitals must carefully consider which staff member is most suitable for this role*’. In our study the three items which are known mainly to the surgeon (critical steps, length of surgery and anticipated blood loss) were poorly completed overall (all compliance less than 60%). The strong statistical association of poorer compliance with a nurse-led process rather than anaesthetist-led is stark, and may be due to lower status of nurses with less role flexibility in China [26] and therefore an unwillingness to interrupt the surgeon. The PUMCH study explicitly attempted to establish the circulating nurse to lead the SSC but this failed [6]. A higher profile for nurse confidence/assertiveness training as an adjunct to patient safety might shift this dynamic.

Completion rates for items of the ‘*sign-out*’ component which remain part of the MoH SSC are superior (98%) to those reported in China before [6]. However, ‘*equipment problems to be addressed*’ were only discussed in 66% of cases and ‘*concerns for recovery and patient management*’ in 28%; the lack of an anaesthetist at ‘sign-out’ at two sites may have had an impact on this observation.

All SSCs were signed-off as showing every item was completed, however no item showed 100% compliance at all sites. This is similar to findings in some other countries. One survey in the United States showed that nearly 40% of respondents simply checked off boxes ahead of time [27]. The overall rate of compliance for all items of the WHO SSC and across all five sites was 79.8%, similar to the approximately 80% ‘completion rate’ observed in 2015 across four sites recorded in a previous large Chinese study [6]. However, one hospital (site D) had a significantly better compliance-rate than all the others. At site D policing of operating room checklist behaviour was much more robust than any of the other hospitals. Members of a supervisory theatre group were devolved the authority to ‘spot-check’ compliance at any time. Lack of checklist engagement held the risk of imposition of a monetary penalty (equivalent to about 15 US$) to be subtracted from base-salary and represented poor behaviour to be raised during annual staff appraisal. At site D the stimulus for formation of the inspection group was a voluntary effort to improve auditable safety programmes in the hospital, driven by a desire to gain extra ranking points for the hospital in national hierarchy tables. Together with site E, site D had the highest inter-professional participation rates in all sections of the SSC.

The WHO Guidelines for Safe Surgery 2009 affirms that ‘*checklists must be tested in their clinical setting to affirm their value*’. In China we found that all items within the WHO SSC were being discussed to varying degrees in all five hospitals we surveyed. Those which are omitted in the China SSC were discussed over half the time, suggesting a deeper recognition of the relevance of those interventions to patient safety.

Table 6 shows that the majority of the operative procedures were carried out without significant peri-operative adverse issues. Although site D had obvious problems with instrument tray completeness during the period of observation, all cases included discussion about equipment problems during the ‘*sign-out*’ component. The value of the SSC may be clearly seen in the care taken to identify and redress instrument failures prior to starting surgery at site D.

We used purposive and snowball sampling so as to ascertain professionals from every Province in Mainland China. However this valid sociological method to sample hard-to-reach groups is a non-random method and has inherent selection biases which are uncontrolled. The results of our survey on attitude may therefore not represent all views or even representative views of operating room staff and surgeons although we have no reason to believe they do not. Furthermore, sampling of compliance followed established protocol elsewhere in which a single operating room in each centre was chosen for observation. Selection bias in terms of case- and staff-mix may have resulted.

## Conclusion

The WHO SSC remains a powerful tool for surgical patient safety in China. Cultural changes in nursing assertiveness and surgeon-led teamwork and checklist ownership are the key elements for improving compliance. Standardised audits are helpful to monitor and ensure checklist compliance. Further research in support of the optimum number and content of MoH SSC items would be welcome.

## Data Availability

The datasets generated and/or analysed during the current study are not publicly available but are available from the corresponding author on reasonable request.

## Abbreviations

MoH: Chinese Ministry of Health
SSC: Surgical Safety Checklist
WHO: World Health Organisation
PUMCH: Peking Union Medical College Hospital
SAQ: Safety Attitudes Questionnaire
